# Clinical benefits of routine examination and synchronous repair of occult inguinal hernia during laparoscopic peritoneal dialysis catheter insertion: a single-center experience

**DOI:** 10.1007/s10029-020-02364-7

**Published:** 2021-02-06

**Authors:** H.-W. Kou, C.-N. Yeh, C.-Y. Tsai, J.-T. Hsu, S.-Y. Wang, C.-W. Lee, M.-C. Yu, T.-L. Hwang

**Affiliations:** Division of General Surgery, Department of Surgery, Chang Gung Memorial Hospital, Linkou Medical Center, Guishan, Taoyuan Taiwan

**Keywords:** Peritoneal dialysis catheter insertion, Laparoscopic examination, Occult inguinal hernia, Synchronous hernia repair

## Abstract

**Purpose:**

Occult inguinal hernias (IH) predispose peritoneal dialysis (PD) patients to the symptomatic IH formation after starting PD, which may cause complications. We conducted a retrospective study to assess the benefit/risk profile of routine laparoscopic examination for occult IH (RLEOH) with a synchronous repair in patients receiving PD catheter placement.

**Methods:**

432 patients were enrolled in this study. Patients with an internal hernia sac at all sizes were deemed to have occult IH. We retrospectively reviewed data including demographic characteristics and operative details. We also measured incidence rates of symptomatic IH, metachronous IH repair, and catheter survival over a follow-up period after starting PD.

**Results:**

These patients were classified into the RLEOH group (*n* = 365) and the non-RLEOH group (*n* = 67). The RLEOH group was subdivided into occult IH with a synchronous repair (*n* = 17; the subgroup A), no occult IH (*n* = 339; the subgroup B), and occult IH without a synchronous repair (*n* = 9; the subgroup C). The incidence rates of symptomatic IH developed after staring PD in subgroups A, B, and C were 0, 5.6, and 22.2%, respectively, whereas that in the non-RLEOH group was 13.4%. The RLEOH group had a reduced hazard ratio for metachronous IH repair compared with the non-RLEOH group (HR = 0.426; 95% CI 0.195–0.930, *p* = 0.032). None of our patients suffered from herniorrhaphy-related complications.

**Conclusion:**

RLEOH with a synchronous repair during PD catheter insertion confers clinical benefits in reducing the risk of developing IH after starting PD and the need for a metachronous repair. This is a safe and reasonable approach.

**Supplementary Information:**

The online version contains supplementary material available at 10.1007/s10029-020-02364-7.

## Introduction

Continuous ambulatory peritoneal dialysis (PD) has been increasingly used for renal replacement therapy in end-stage renal disease because it has several advantages over hemodialysis such as ease of use and relatively low cost [1–3]. It is manually performed with injection of the dialysate into the peritoneal cavity via a trans-abdominal catheter 3–4 times every day at home [2, 3]. However, the injection of the dialysate inevitably increases the intra-abdominal pressure which may contribute to hernia formation, particularly in patients with abdominal weakness or defects [3–5]. Hernias in patients on PD are clinically important because it may cause complications such as dialysate leakage, hernia incarceration, strangulation, bowel obstruction, and peritonitis [4–8]. Hernia formation may lead to technique failure and conversion from PD to hemodialysis [1, 8, 9]. For these reasons, the guidelines recommend that surgeon should detect and repair preexisting hernias in patients who have chosen PD as their dialysis modality [10]. Preexisting hernias in these patients can be repaired before or at the time of PD catheter insertion [4, 5, 11–14].

A high prevalence of inguinal hernias has been reported in patients before or undergoing PD [4–6, 11, 12] and thus it is important to check preexisting hernias at this anatomic location before starting PD. Symptomatic inguinal hernias are relatively easy to identify, but the diagnosis of occult or asymptomatic inguinal hernias can be challenging even with pre-operative radiologic assessments [15, 16]. Failure to detect occult inguinal hernias may lead to several complications after starting PD [17–19] and the requirement of a metachronous repair. The application of laparoscopy for PD catheter insertion has recently increased [10, 20]. It has been suggested that this technique allows the identification of occult inguinal hernias during PD catheter insertion [5, 10]. However, no comparative study has been conducted to provide evidence of clinical benefits supporting this recommendation.

In this retrospective cohort study, we aimed to assess the benefit/risk profile of routine laparoscopic examination for occult inguinal hernia (RLEOH) with a synchronous repair when possible in patients receiving PD catheter placement. We compared incidence rates of symptomatic hernia, metachronous hernia repair, and catheter survival over a follow-up period after starting PD between the RLEOH and non-RLEOH groups.

## Methods

### Patient population

This study was approved by the Institutional Review Board of Chang Gung Memorial Hospital, Taiwan and informed consent was waived for this retrospective study according to our institutional guideline. From January 2013 to October 2019, patients initiated PD in Linkou Chang Gung Memorial Hospital were identified and their data were extracted from the institution’s database. Patients with no previous record of symptomatic inguinal hernia were identified. Patients who had their PD catheter insertions in other hospitals, an age ≤ 18-year-old or early withdraw from PD within 3 months were excluded.

### Definitions and data collection

**Fig. 1:**
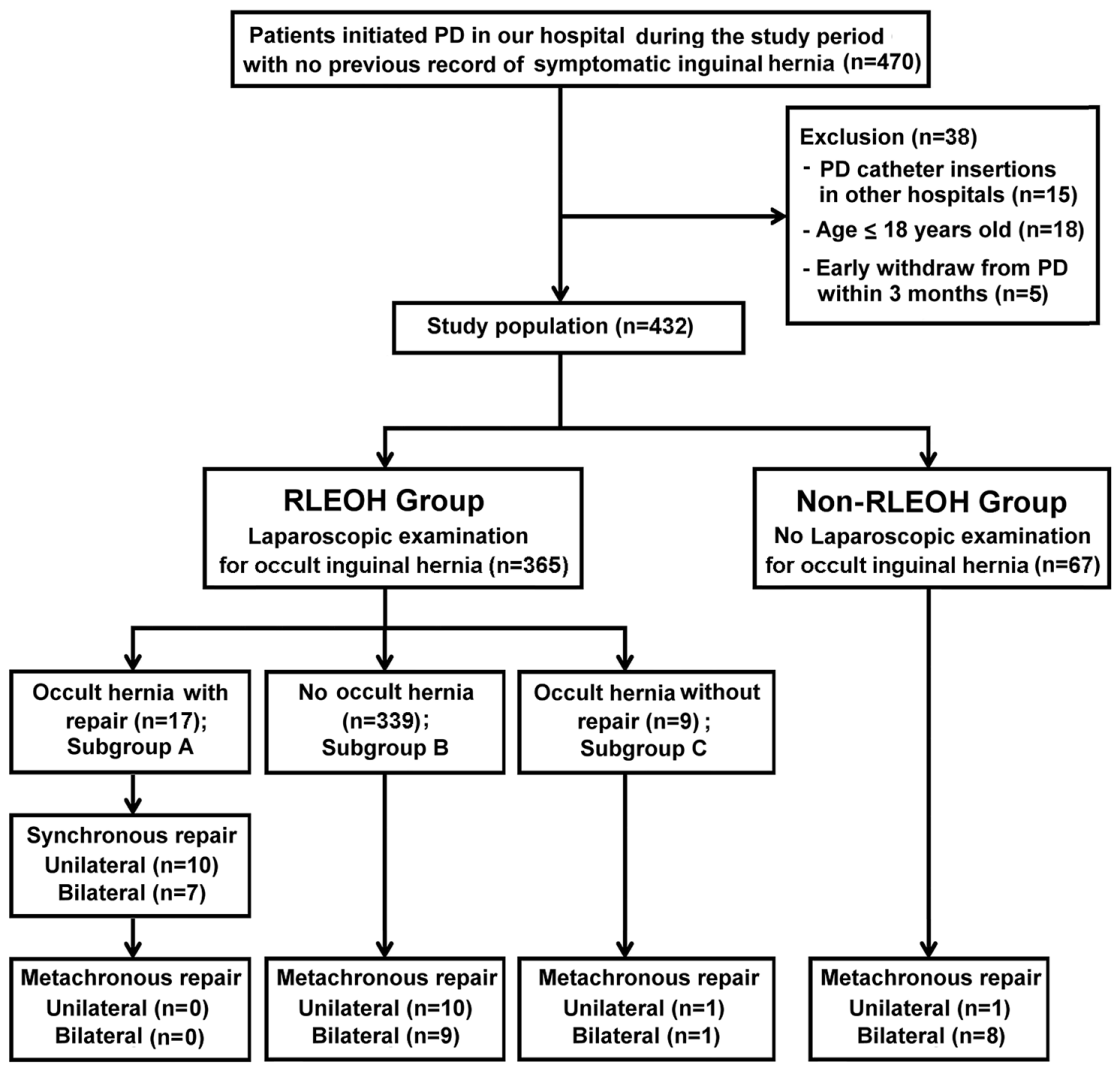
Flowchart of patient disposition. *PD* peritoneal dialysis, *RLEOH* routine laparoscopic examination for occult inguinal hernia, *Non-RLEOH* no routine laparoscopic examination for occult inguinal hernia

Patients who received and did not receive routine examination for occult inguinal hernia on both anatomic sides during laparoscopic PD catheter insertion were classified as the RLEOH and non-RLEOH group, respectively (Fig. 1). The choices for performing or not performing RLEOH were based upon surgeons’ decisions. Synchronous hernia repairs were performed in patients in the RLEOH group if occult inguinal hernia with an internal hernia sac at all sizes presented during the laparoscopic examination and if informed consents were obtained prior to surgery. We retrospectively reviewed data including demographic characteristics, preoperative laboratory examinations, and operative details. Event-free survival was defined as time form PD catheter insertion to metachronous hernia repair or the date of the follow-up without event. Overall catheter survival was defined as time from PD catheter insertion to discontinuation of PD or the date of the follow-up without event. Herniorrhaphy-related surgical complications were defined as wound infection, bleeding, dialysate leakage, hernia recurrence or mortality within 30 days after synchronous or metachronous hernia repair.

### Statistical analysis

The Kolmogorov–Smirnov test was used to check the distribution of the continuous variables. Continuous variables were compared using the Student *t* test in the cases of the normal distribution of data or Mann–Whitney *U* test in the cases of the non-normal distribution of data and presented as mean ± standard deviation (SD) or median with interquartile ranges (IQR), respectively. Pearson’s *χ*^2^ test or Fisher’s exact test was used to compare the categorical variables which are presented as frequency and percentages. The Kaplan–Meier curve analysis followed by the log-rank test was used to depict the time-to-event data and to examine differences between the two study groups. Subsequently, hazard ratios (HRs) and 95% confidence intervals (CIs) were calculated by univariate Cox proportional hazards regression model. All statistical analyses were performed using IBM SPSS Statistics 21 (IBM Corporation, Software Group, Somers, NY, USA). Two-tailed *p* values of less than 0.05 were considered statistically significant for all analyses.

## Results

During the study period, 470 patients initiated PD in our hospital. After exclusion, a total of 432 patients (217 males and 215 females) with no previous record of symptomatic inguinal hernia were enrolled in this study. Mean age of our study population was 51.4 ± 15.3 years (range 19–95 years); mean follow-up time was 33.5 ± 20.8 months (range 3.4–87.9 months). These patients were classified into the RLEOH group (*n* = 365, 84.5%) and the non-RLEOH group (*n* = 67, 15.5%) according to whether or not they had received routine laparoscopic examination (Fig. 1). Table S1 (Supplementary Data) and Table 1 show comparisons of clinical characteristics and operative details, respectively, between these two study groups. As shown, there were no significant differences in age, gender, cause of end-stage renal disease, body mass index, preoperative laboratory examination, and operative time between two study groups. The patients in the non-RLEOH group had a higher rate of history of previous abdominal surgery (23.9 vs. 10.7%, *p* = 0.003), open PD insertion (11.9 vs. 0.0%, *p* < 0.001), and intraabdominal adhesion (20.9 vs. 9.3%, *p* = 0.006).

**Table 1:** Operative details of the two study groups

	RLEOH group (*n* = 365)	Non-RLEOH group (*n* = 67)	*p* value
Operative time (mins)	75 (65–87)	73 (60–91)	0.491
Method			
Laparoscopic	365 (100.0)	59 (88.1)	< 0.001
Open	0 (0.0)	8 (11.9)	
Intraabdominal adhesion	34 (9.3)	14 (20.9)	0.006
Presence of occult inguinal hernia	26 (7.1)	–	
Time from PD catheter insertion to metachronous hernia repair (days)	181 (78–679)	159 (78–284)	0.667
Metachronous symptomatic hernia	21 (5.8)	9 (13.4)	0.034

Data are presented as *n* (%) or median (interquartile ranges)

*RLEOH* routine laparoscopic examination for an occult inguinal hernia during peritoneal dialysis catheter placement, *PD* peritoneal dialysis

In the RLEOH group, 17 patients were deemed to have occult inguinal hernia and received a synchronous hernia repair during their PD catheter insertion (Fig. 1; the subgroup A). Among them, 7 patients had bilateral herniorrhaphy and 10 had unilateral herniorrhaphy. There was no delay in the time from PD catheter insertion to the first course of PD in patient received synchronous hernia repair (*n* = 17) compared to other patients (*n* = 415) in this study population [11 days (9.5–14.5) vs. 10 days (8–12); *p* = 0.126]. Additionally, in the RLEOH group, 339 patients were deemed to have no occult inguinal hernia (Fig. 1; the subgroup B), while 9 patients had confirmed occult inguinal hernia without a synchronous repair due to a lack of informed consent (Fig. 1; the subgroup C). Patients in the subgroup C (*n* = 9) did not have complications except a relatively high incidence of symptomatic inguinal hernia established during the follow-up period. The incidence rates of symptomatic inguinal hernia, either unilateral or bilateral, developed during the follow-up period after staring PD in subgroups A, B, and C were 0, 5.6, and 22.2%, respectively, whereas that in the non-RLEOH group was 13.4%.

**Fig. 2:**
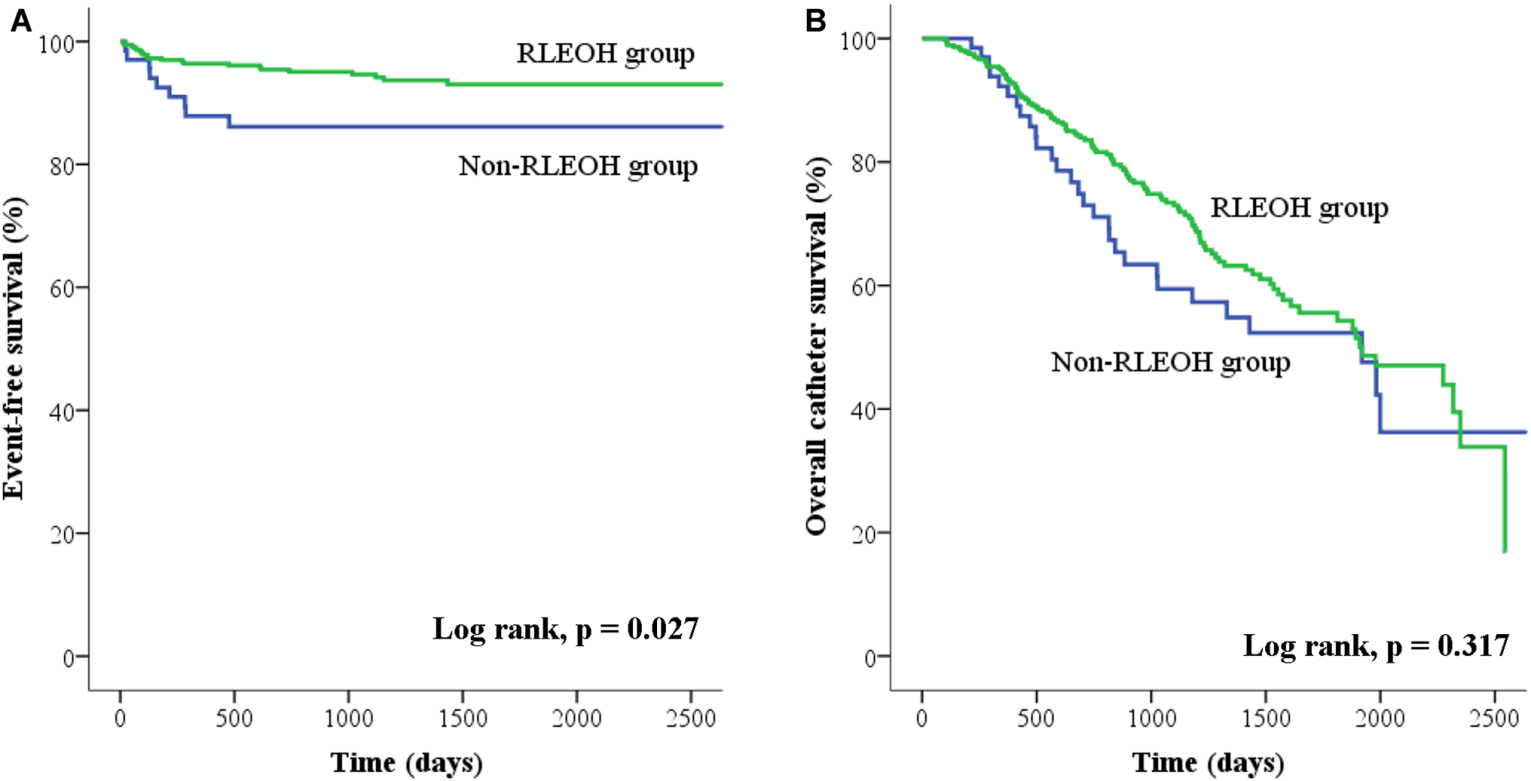
Kaplan–Meier curves of the event (metachronous hernia repair) free (**a**) and overall catheter survival (**b**) for patients in the RLEOH group (*n* = 365; green line) versus the non-RLEOH group (*n* = 67; blue line). *RLEOH* routine laparoscopic examination for occult inguinal hernia, *Non-RLEOH* no routine laparoscopic examination for occult inguinal hernia

Once symptomatic inguinal hernia was diagnosed, all patients were subjected to a metachronous hernia repair. The numbers of patients who received unilateral or bilateral metachronous hernia repair in subgroups A, B, and C as well as in the non-RLEOH group are indicated in Fig. 1. Particularly, the anatomical sides of the metachronous hernia repair in 2 patients of the subgroup C (Fig. 1) were the same as those of occult inguinal hernia identified initially during the laparoscopic examination. Overall, the percentage of the metachronous hernia repair was significantly higher in the non-RLEOIH group compared to the RLEOH group (13.4 vs. 5.8%, *p* = 0.034; Table 1). The median time from PD catheter insertion to the metachronous hernia repair was not significantly different between two study groups (*p* = 0.667; Table 2). The Kaplan–Meier curve analysis (Fig. 2a) followed by the log-rank test revealed a significant difference in the event (metachronous hernia repair) free survival over time between two study groups (*p* = 0.027). Further analysis indicated that patients in the RLEOH group had a reduced HR for metachronous hernia repair compared with those in the non-RLEOH group (HR = 0.426; 95% CI 0.195–0.930, *p* = 0.032). The overall catheter survival over rime (Fig. 2b) was not significantly different between two study groups (*p* = 0.317). Patients with a history of previous abdominal surgery (HR = 1.09; 95% CI 0.38–3.12, *p* = 0.875) or intraabdominal adhesion (HR = 2.04; 95% CI 0.83–4.98, *p* = 0.120) did not have an increased risk of metachronous hernia repair compared to those without. Table 2 shows clinical demographics and characteristics of patients who received synchronous or metachronous hernia repair (*n* = 47) and patients who did not need repair (*n* = 385) in this study. As shown, patients in the hernia repair group had a higher percentage of male as compared to those in the non-repair group. There were no significant differences in other variables between the hernia repair and non-repair group (Table 2).

**Table 2:** Clinical demographics and characteristics of patients who received inguinal hernia repair and who did not need repair in this study

	No need for hernia repair (*n* = 385)	With synchronous or metachronous hernia repair (*n* = 47)	*p* value
Age (years)	51.3 ± 15.7	49.5 ± 14.6	0.448
Gender			
Male	180 (46.3)	37 (78.7)	< 0.001
Female	205 (53.2)	10 (21.3)	
Body mass index (kg/m^2^)	23.9 ± 4.6	24.6 ± 4.5	0.319
Cause of ESRD			0.253
Glomerulonephritis	137 (35.6)	19 (40.4)	
Diabetes mellitus	130 (33.8)	19 (40.4)	
Hypertensive	32 (8.3)	4 (8.5)	
Obstructive nephropathy	11 (2.9)	0 (0.0)	
Polycystic renal disease	7 (1.8)	2 (4.3)	
Unknown	68 (17.7)	3 (6.4)	
History of abdominal surgery	50 (13.0)	5 (10.6)	0.648
Preoperative laboratory exam			
Creatinine (mg/dL)	10.2 (8.6–12.8)	10.6 (8.7–13.2)	0.547
eGFR (mL/min/1.73 m^2^)	4.5 (3.7–5.8)	4.6 (3.9–6.1)	0.286
Albumin (g/dL)	3.5 ± 0.6	3.7 ± 0.6	0.103
Hemoglobin (g/dL)	8.8 (8.0–9.6)	8.9 (8.2–9.9)	0.393
Platelet count (1000/μL)	201.1 ± 73.7	205.1 ± 63.8	0.725
Sodium (mEq/L)	137 (134–140)	138.5 (136–140)	0.114
Potassium (mEq/L)	4.3 ± 0.7	4.4 ± 0.7	0.199
Calcium (mg/dL)	8.1 (7.4–8.6)	7.9 (7.2–8.7)	0.435
Phosphorus (mg/dL)	6.2 (5.0–7.6)	6.2 (5.0–7.1)	0.325

Data are presented as mean ± standard deviation, *n* (%) or median (interquartile ranges)

*ESRD* end-stage renal disease, *eGFR* estimated Glomerular filtration rate, *ALT* aspartate transaminase

In this study, the open technique was employed as the approach for synchronous (*n* = 17) or metachronous (*n* = 30) hernia repair. Among the patients with synchronous hernia repair, 15, 1, and 1 patients received McVay repair, Lichtenstein tension-free mesh repair, and Bassini repair, respectively. Among the patients with metachronous hernia repair, 25, 2, 2, and 1 patients received McVay repair, Lichtenstein tension-free mesh repair, Bassini repair, and high ligation of the indirect sac, respectively. As a group, the operation time for patients with additional synchronous hernia repair (123 min, IQR = 90–145; *n* = 17) was significantly (*p* < 0.001) longer than that for patients with usual PD catheter placement (75 min, IQR = 64–86; *n* = 415). No herniorrhaphy-related surgical complications occurred in the patients who received synchronous or metachronous hernia repair in this study. Also, patients who had confirmed occult inguinal hernia without a synchronous repair (Fig. 1; the subgroup C, *n* = 9) did not have complications except a relatively high incidence of symptomatic inguinal hernia established during the follow-up period.

## Discussion

In this retrospective study, our results clearly show that patients who received RLEOH had a reduced risk of metachronous inguinal hernia repair compared to those in the non-RLEOH group. This benefit is mainly due to the fact that a majority (65.4%) of the patients in the RLEOH group who had occult inguinal hernia received a synchronous hernia repair (the subgroup A). Importantly, none of these patients developed symptomatic inguinal hernias during the follow-up period after starting PD, thus reducing the need for a metachronous surgery. This favorable outcome is in contrast to the finding that more than one-fifth of the patients (22.2%) who had confirmed occult inguinal hernia without a synchronous repair (the subgroup C) eventually developed symptomatic inguinal hernias after starting PD. The prevalence of symptomatic inguinal hernia in patients undergoing PD ranges from 11 to 33% [4, 6, 11] and the incidence (13.4%) in the non-RLEOH group is within this range. Our patients who had no occult inguinal hernia (the subgroup B) appear to have a relatively low incidence (5.6%) of newly developed inguinal hernia during PD, another favorable outcome presumably resulting from RLEOH. Collectively, these results suggest the benefits of RLEOH with a synchronous hernia repair in reducing the incidence of developing a symptomatic inguinal hernia, the need for a metachronous repair, and the risk of complications associated with hernia formation or additional surgery in patients undergoing PD. On the other hand, no difference in the overall catheter survival over time between two study groups was found. In addition, the procedure of synchronous hernia repair during the PD catheter insertion did not delay the first course of PD and did not cause any herniorrhaphy-related surgical complication in our patients, although this procedure did take a longer operation time as compared to the usual PD catheter insertion. Thus, this approach seems to be reliable and safe, a finding that is in good agreement with those in the study of symptomatic hernia repair and simultaneous PD insertion [12–14].

It has been reported that male, advanced age, high body mass index, polycystic kidney disease, long duration of dialysis, and history of abdominal surgery are risk factors for hernia development in PD patients [9, 21–24]. However, except the percentage of male, we found no differences in these variables between patients who received synchronous or metachronous hernia repair and patients who did not need repair. We found patients in the hernia repair group indeed had a higher percentage of male, a finding that is consistent with that reported by other investigators [9]. Our patients in the non-RLEOH group did have higher incidences of history of abdominal surgery and intraabdominal adhesion; the former is a risk factor of hernia formation in patients on PD [22, 23], whereas the role of the latter is undefined. Whatever their potential impacts, these two factors did not increase the risk of metachronous hernia repair in our patient population as revealed by our regression analyses.

The definition of occult inguinal hernia has not been well defined in the literature. In 2018, the international guidelines defined an occult hernia as an asymptomatic hernia that is not detectable by physical examination [26]. van den Heuve et al. [27] believed that occult inguinal hernias with an internal sac of no more than 2 cm in a general population are unlikely to allow any herniation. In this study, we defined occult inguinal hernia if a hernia sac at all sizes was presented during the laparoscopic examination; this is because PD patients are a population with high risk. Our study shows a prevalence of 7.1% of occult inguinal hernia in patients who underwent thorough RLEOH. There has been no study reporting the incidence rate in this patient population, but few cases have been reported whose occult inguinal hernias were missed by routine examinations, but later were confirmed during PD [18, 19, 25]. Symptomatic inguinal hernias are relatively easy to identify with pre-operative physical assessments. When patients present vague signs of inguinal hernias, radiologic modalities such as ultrasonography and magnetic resonance imaging can be used for the diagnosis [5]. However, these radiologic assessments would not be routinely performed in PD patients with no previous record and no symptoms of inguinal hernias (occult or asymptomatic inguinal hernias). The diagnosis of occult inguinal hernias remains challenging because preoperative imaging assessments cannot reliably exclude this entity due to a high false-negative rate [15, 16]. Accordingly, occult inguinal hernias can be more precisely identified by careful laparoscopic examination of the abdominal cavity in patients receiving PD catheter placement.

An occult inguinal hernia may represent an abdominal defect that is associated with abnormal connective tissue alterations [28] and is vulnerable to the impact of increased intraabdominal pressure occurred in patients undergoing PD [3–5]. This may predispose these patients to the development of symptomatic inguinal hernias. Indeed, we observed 22.2% of the patients whose occult inguinal hernias were not synchronously repaired, but later required metachronous hernia repairs on the same anatomical sides. Similarly, in two studies performing laparoscopic repair of unilateral diagnosed inguinal hernias [27, 29], the investigators also reported that 21% or 28.6% patients who had occult inguinal hernias on the contralateral side became symptomatic and required metachronous repairs. Our finding regarding the high recurrence rate suggests that all occult inguinal hernias, regardless their sizes, should be repaired when found during PD catheter insertion.

In this study, we employed the open technique as the approach for synchronous or metachronous hernia repair. Either open or laparoscopic technique has been employed as the approach for concomitant hernia repair and PD catheter placement; both approaches were reported to be reliable and safe [5]. So far, no comparative study has been conducted to investigate which one is superior to another. However, the use of the laparoscopic technique in this setting bears a risk of damaging the peritoneal membrane, a concern that requires to be considered in PD patients [5].

There are no guidelines regarding the timing to commence or continue PD after synchronous or metachronous hernia repair. The decision of this timing should be based upon at least three major considerations [5]. First, sufficient postoperative time should be allowed for proper healing to avoid dialysate leakage from hernia repair site. Second, the duration for PD to be withheld postoperatively depends on the residual renal function of patients as judged by nephrologists. Third, whether adequate strategies of PD treatment are applied to avoid postoperative dialysate leakage from hernia repair site. Our institution uses an approach of urgent-start PD that involves the initiation of PD therapy earlier than 2 weeks after PD catheter insertion [30]. In our cases with synchronous hernia repair, we tended to allow two more days if possible for proper healing after surgery. In this study, the medium times from PD catheter insertion to the first course of PD in patients who received and who did not receive synchronous hernia repair were around 11 and 10 days, respectively. In our cases with metachronous hernia repair, PD treatment could be restarted 3 days after surgery with a strategy of low-volume high-frequency exchanges [5].

There were some limitations to the current study. First, this is a retrospective study with a relatively small sample size of patients from a single institution. Future prospective investigations with a larger sample size for a longer follow-up duration are warranted. The second limitation is the lack of certain data available in each patient, such as the amount of dialysate injected per day and intra-abdominal pressure during the follow-up period; the changes in these variables may potentially contribute to hernia formation (3–5). Third, the criteria for identification of occult inguinal hernias by RLEOH may vary over time or among different surgeons, which may influence the diagnosis. However, this was largely avoided by the situation that most of the cases (83%) were performed or supervised by a senior surgeon (T.L.H). Fourth, there is a notable difference in the number of patients with metachronous repair (*n* = 30) in relation to those with synchronous repair (*n* = 17); this produces certain difficulty to make a real assessment of these two procedures.

In conclusion, our results suggest that RLEOH with a synchronous repair during PD catheter insertion may confer clinical benefits in reducing both the risk of developing symptomatic inguinal hernias after starting PD and the need for a metachronous surgery and this approach is safe. We advocate that this approach should be set as a strategy in the guidelines. We also urge that the possibility of identifying and simultaneous repair an occult inguinal hernia should be discussed with patients who have chosen PD as their dialysis modality in the preoperative consent.

## Supplementary Information

Below is the link to the electronic supplementary material.

Supplementary file1 (DOCX 17 KB)
