# Supplementary material for: Clinical benefits of routine examination and synchronous repair of occult inguinal hernia during laparoscopic peritoneal dialysis catheter insertion: a single-center experience
Source: Hernia. 2021 Feb 6;25(5):1317–24. doi: 10.1007/s10029-020-02364-7 (PMC8514383; doi:10.1007/s10029-020-02364-7)
Supplement: Supplementary file 1 — Supplementary file1 (DOCX 17 KB) [file 10029_2020_2364_MOESM1_ESM.docx]

Article: Clinical Benefits of Routine Examination and Synchronous Repair of Occult Inguinal Hernia during Laparoscopic Peritoneal Dialysis Catheter Insertion: A Single Center Experience

Authors: Hao-Wei Kou, Chun-Nan Yeh, Chun-Yi Tsai, Jun-Te Hsu, Shang-Yu Wang, Chao-Wei Lee, Ming-Chin Yu, Tsann-long Hwang

**Supplementary Data**

**Table S1. Clinical demographics and characteristics of the two study groups**

|  | | **RLEOH group (n=365)** | **Non-RLEOH group (n=67)** | **p value** |
| --- | --- | --- | --- | --- |
| **Age (years)** | | 51.5 ± 15.5 | 51.0 ± 14.3 | 0.811 |
| **Gender** | **Male** | 177 (48.5) | 40 (59.7) | 0.092 |
|  | **Female** | 188 (51.5) | 27 (40.3) |  |
| **Body mass index (kg/m^2^)** | | 23.9 ± 4.5 | 24.6 ± 4.5 | 0.226 |
| **Cause of ESRD** | |  |  | 0.878 |
| **Glomerulonephritis** | | 134 (36.7) | 22 (32.8) |  |
| **Diabetes mellitus** | | 124 (34.0) | 25 (37.3) |  |
| **Hypertensive** | | 31 (8.5) | 5 (7.5) |  |
| **Obstructive nephropathy** | | 8 (2.2) | 3 (4.5) |  |
| **Polycystic renal disease** | | 8 (2.2) | 1 (1.5) |  |
| **Unknown** | | 60 (16.4) | 11 (16.4) |  |
| **History of abdominal surgery** | | 39 (10.7) | 16 (23.9) | **0.003** |
| **Preoperative laboratory exam** | |  |  |  |
| **Creatinine (mg/dL)** | | 10.3 (8.8 – 13.0) | 10.1 (8.0 – 12.9) | 0.304 |
| **eGFR (mL/min/1.73 m^2^)** | | 4.5 (3.6 – 5.6) | 4.8 (3.9 – 6.4) | 0.104 |
| **Albumin (g/dL)** | | 3.5 ± 0.6 | 3.6 ± 0.5 | 0.657 |
| **Hemoglobin (g/dL)** | | 8.8 (8.1 – 9.6) | 8.8 (7.9 – 9.9) | 0.594 |
| **Platelet count (1000/μL)** | | 193 (152 – 243) | 194 (153 – 240) | 0.793 |
| **Sodium (mEq/L)** | | 137 (134 – 140) | 138 (134 – 140) | 0.841 |
| **Potassium (mEq/L)** | | 4.3 (3.9 – 4.8) | 4.3 (3.8 – 4.9) | 0.868 |
| **Calcium (mg/dL)** | | 8.1 (7.3 – 8.6) | 8.3 (7.7 – 8.6) | 0.234 |
| **Phosphorus (mg/dL)** | | 6.3 (5.0 – 7.6) | 6.3 (4.8 – 7.1) | 0.467 |

RLEOH, routine laparoscopic examination for occult inguinal hernia during peritoneal dialysis catheter placement; ESRD, end stage renal disease; eGFR, estimated Glomerular filtration rate; ALT, aspartate transaminase. Data are presented as mean ± standard deviation, n (%) or median (interquartile ranges).
